# Foot and ankle mobility and the frontal plane projection angle in asymptomatic controls

**DOI:** 10.1186/1757-1146-8-S2-O43

**Published:** 2015-09-22

**Authors:** Narelle Wyndow, Amy De Jong, Krystal Rial, Kylie Tucker, Natalie Collins, Bill Vicenzino, Trevor Russell, Kay Crossley

**Affiliations:** 1 The University of Queensland, Division of Physiotherapy, School of Health & Rehabilitation Sciences, St. Lucia Queensland 4072, Australia; 2 The University of Queensland, School of Biomedical Sciences, St. Lucia Queensland 4072, Australia; 3 Department of Mechanical Engineering, Melbourne School of Engineering, University of Melbourne, Melbourne Victoria 3010, Australia

**Keywords:** Foot mobility, ankle mobility, frontal plane Projection angle

## Introduction

The frontal plane projection angle (FPPA) is a measure of the degree of dynamic knee valgus during functional tasks such as the single leg squat. An increased FPPA is associated with patellofemoral pain and is indicative of knee valgus during other tasks such as running. Therefore, it is important to determine factors that can contribute to FPPA in order to develop treatment strategies for people with (or at risk of) PFJ pain.

## Methods

The FPPA was measured during a single leg squat (FPPASq), and single leg hop (FPPAhop) in 30 healthy controls aged 18-50 years. The FPPA was determined as the valgus (or varus) angle at the knee formed by lines connecting the anterior superior iliac spine, the midpoint of the femoral condyles and the midpoint of the malleoli at the deepest part of the squat, or at the point of full foot contact during the hop. Knee valgus excursion was determined as the difference in FPPA from the start to the bottom of the single leg squat (FPPASqexc). Foot mobility was quantified as the difference in dorsal midfoot arch height, or midfoot width, between non-weight bearing and bilateral weight bearing (50% total body weight), at 50% of the total foot length. Ankle joint range was measured as the maximum distance between the longest toe and the wall during a knee-to-wall lunge in the sagittal plane.

## Results

Higher midfoot width mobility was associated with a greater FPPASq (p = < .001), FPPAhop (p = .008), and FPPASqexc (p = < .001). Smaller ankle joint dorsiflexion range was associated with an increased FPPASq (p = .004), FPPAhop (p = .002), and FPPSSqexc (p = .003). Midfoot height mobility was only associated with an increased peak FPPASq (p = .002).

## Discussion

The findings of this study indicate that foot and ankle mobility is associated with an increased FPPA during the SLSq and SLH in healthy individuals. Specifically, an increased midfoot width mobility, or reduced ankle joint dorsiflexion range, are both associated with increased FPPA. These two factors require consideration in the clinical management of knee related disorders such as patellofemoral pain.

